# Community Intervention Self-Efficacy Scale for Parent Leaders (CONNECTED): Parents’ Empowerment to Prevent Adolescent Alcohol Use

**DOI:** 10.3390/ijerph17134812

**Published:** 2020-07-04

**Authors:** Daniel Lloret, Elena Gervilla, Montse Juan, Yasmina Castaño, Claudia R. Pischke, Florence Samkange-Zeeb, Fernando Mendes

**Affiliations:** 1 European Institute of Studies on Prevention, 07003 Palma, Spain; daniel.lloret@alu.umh.es (D.L.); mjuan@irefrea.org (M.J.); ycastano@irefrea.org (Y.C.); 2 Department of Health Psychology, Miguel Hernandez University, 03202 Alicante, Spain; 3 Balearic Islands Health Research Institute, 07120 Palma, Spain; 4 Department of Psychology, University of the Balearic Islands, 07122 Palma, Spain; 5 Institute of Medical Sociology, Centre for Health and Society, Medical Faculty, Heinrich Heine University Duesseldorf, 40225 Duesseldorf, Germany; Claudia.Pischke@hhu.de; 6 Leibniz Institute for Prevention Research and Epidemiology—BIPS, 28359 Bremen, Germany; samkange@leibniz-bips.de; 7 Instituto Europeu para o Estudo dos Factores de Risco em Crianças e Adolescentes, 3030-218 Coimbra, Portugal; irefrea.pt@gmail.com

**Keywords:** empowerment, parents, family, leadership, assessment, environment, community participation

## Abstract

Empowering parents by actively engaging them in environmental prevention strategies is a promising approach that only a few programs use. Evidence suggests that when families and the wider community are engaged, alcohol prevention is more efficient. However, due to the novelty of this approach, no specific assessment tools for measuring this type of engagement are available. The objective of this study is to design a parental empowerment measurement tool to evaluate parents’ self-efficacy when engaging in environmental and community actions and to analyze its psychometric properties. A total of 132 parents active in in-school parent associations from Spain (*n* = 77; 58.4%) and Portugal (*n* = 55; 41.7%) completed a pencil and paper battery of four questionnaires, including the developed scale COmmuNity iNtervention SElf-Efficacy SCale for ParenT LEaDers (CONNECTED). The scale showed a good reliability and good test-retest stability in a three-month period. The convergent validity with other well-established instruments that assess similar constructs was significant. A preliminary confirmatory factor analysis (CFA) showed an acceptable fit. Environmental prevention supported by families is a promising preventive strategy because the participation and involvement of families is an effective way to address some risks in adolescence; however, new assessment tools are needed in this field. The developed scale could be a first step to identify the areas of need in a community and to monitor the progress and evaluate the outcomes of the preventive interventions implemented.

## 1. Introduction

Environmental prevention comprises interventions that aim to limit the availability of opportunities leading to maladaptive behavior and promote the availability of healthier opportunities through policies, restrictions, and actions [1]. Family associations and particularly in-school parent associations are a key target group for launching, carrying out, and monitoring strategies and actions to identify and restrict threats and promote opportunities for healthy changes at the community level. Therefore, empowering the leaders of parent organizations and, in turn, families to enhance community engagement by involving them in decision-making and in the planning, design, governance, and delivery of interventions constitutes a powerful environmental preventive strategy for addressing maladaptive behaviors such as risky alcohol use among adolescents.

Empowerment has been defined as a multidimensional process by which individuals and groups acquire a better knowledge and control over their lives by expressing their needs and their concerns; devising strategies for involvement in decision-making; and achieving the political, social, and cultural competence to meet those needs [2]. From a more psychological point of view, empowerment is the sense of ownership of one’s life [3] or individual coping mechanisms, confidence, and self-esteem, leading to the ability to take control and make decisions [4]. In other words, empowerment is the psychological ability to obtain an internal locus of control, self-efficacy, and the skills to achieve goals set by oneself and perceiving life as being full of personal and social opportunities [5].

Three levels of empowerment have been suggested [6]: (a) individual or psychological empowerment (the comprehension and defence of one’s rights and responsibilities and beliefs, such as self-efficacy, locus of control, and self-esteem); (b) organizational empowerment (aimed to increase the actions of members of organizations); and (c) community empowerment (actions made by a group of people with the aim to improve community life). Using proactive actions and advocacy, people can control their own environment and families can become active agents within a larger community.

Parental empowerment is defined as the knowledge, skills, and resources that allow parents to have positive control over their lives [7]. Empowerment allows each person to make decisions about their family, organizations, and society and is a dynamic state that depends on diverse life situations and occurrences, as well as available networks, services, and the society [8,9]. Families are systems with their own social networks and have the right to choose their own services and levels of engagement [10,11]. Parental empowerment has also been found to improve parenting resources and self-efficacy; reduce parental stress; strengthen parental engagement in childcare; produce better behavior in adolescents; and positively influence their psychosocial, physical, verbal, and social development [12].

Due to its dynamic nature, assessing empowerment as a process is a challenging task. It is mostly measured using qualitative methods, although it includes intrapersonal (self-efficacy and control beliefs), interactional (social environment), and behavioral components (actions to have control) when regarded as a target or achievement [13]. However, to date parent empowerment evaluation tools are mainly designed to assess how parents face internal family challenges, with a focus on problem solving, decision-making, coping with stress or how to find help or support in professional or parents’ networks. The social environment aspects of empowerment are not included. Considering the role that parents, as an advocacy group, can play in the implementation of promising environmental interventions to influence decision making and improve their social environment, and taking into account the lack of valid evaluation instruments, this article aims to describe the design and analysis of an assessment tool to measure self-efficacy in parents working to engage their communities in actions to prevent adolescent alcohol use.

## 2. Materials and Methods

A total of 132 parents (83.5% women) who were active in 38 public in-school parent associations from the Balearic Islands in Spain (*n* = 77; 58.3%) and the Coimbra region in Portugal (*n* = 55; 41.7%) completed a paper-and-pencil battery of four questionnaires, including the scale developed as part of this study, during parent meetings (all the parents attending the meeting answered the questionnaires). We did not find statistically significant differences in gender by country (*χ*2 = 0.350; *df* = 1; *p* = 0.554).

All the participants were members of parent association boards and were non-randomly recruited through their parents’ associations. Data were collected during the first six months of 2018. The participants took approximately 25 min to answer all the questionnaires.

To identify and compile appropriate items for the different dimensions, we followed a three-phase process as recommended by Boateng et al. [14]:

(1) Creation of an item pool: (a) the establishment of the conceptual structure based on a literature review and the consensus of an expert panel; (b) the development of a pool of items for each theoretical dimension; and (c) the selection of items through a panel of four experts who evaluated the understanding and relevance of each item with respect to its dimension.
(2) Piloting: The pre-testing of questions in a small group of parents (*n* = 10) to examine comprehension, response time, and potential confusing interpretations; sampling and survey administration. The response time was 15 min on average. Since we did not detect misunderstandings during the pilot test, we used the pilot version for a validation analysis.
(3) Psychometric analysis for a reliability, validity, and confirmatory factor analysis.

The scale created, COmmuNity iNtervention SElf-Efficacy SCale for ParenT LEaDers (CONNECTED), was inspired by research on health education and community empowerment carried out by Israel and colleagues [15]. The items, consisting of a 5-point Likert scale (strongly disagree–strongly agree and not capable at all–very capable), conceptualize and measure the perceptions of individual and community control as well as the potential ability to perform actions to improve community health conditions and prevent youth alcohol consumption (see Table 1).

Additionally, we used other scales to measure parents’ actions:

- General self-efficacy scale [16,17,18], which comprises 10 items with a 5-point Likert scale (strongly disagree–strongly agree). The items describe the self-perception of problem solving and the confidence of the participant in their own capacity. The scale had a Cronbach’s reliability of α = 0.87 and a two-halves correlation of 0.88.
- Intention to get involved in community actions was assessed by a four-item scale, as applied by Kasmel and colleagues [19]. Each item was rated on a 5-point Likert scale (strongly disagree–strongly agree), with higher scores representing a higher intention to get involved in the community. The scale had a Cronbach’s reliability of α = 0.96 and a two-halves correlation of 0.95.
- To assess the participants’ behavioral empowerment, we selected the scale used by Speer and colleagues [20]. The scale comprised seven items, which the participants rated on a 5-point Likert scale (from 1–never, to 5–always). The activities detailed in the scale (e.g., signing a petition, attending or organizing meetings, or writing letters) had to have been done during the last three months and represented the usual activity within parents’ association tasks. The scale had a Cronbach’s reliability of α = 0.89 and a two-halves correlation of 0.86.

SPSS version 23.0 [21] and MPlus version 8.1 [22] were used to conduct the quantitative analysis. Descriptive statistics were used to calculate the sample means and standard deviations, skewness and kurtosis statistics for the items. We computed the item-total correlations, scale reliability coefficient (Cronbach’s α), and test-retest reliability (intraclass correlation coefficient), as well as the convergent validity (Spearman’s correlation).

The internal consistency was measured with Cronbach’s alpha coefficients. To analyze the convergent validity, we compared the scores of three measures theoretically related to individual and community self-efficacy: the general self-efficacy scale, intention to get involved in the community scale, and parents’ association participation questionnaire. To explore the relationship between these scores, we used Spearman’s Rho correlation coefficient.

## 3. Results

### 3.1. Item Analysis

A descriptive analysis of the items revealed that the adjusted item-total correlations were above 0.3 in all cases. The skewness statistics had negative values for all the items, and the kurtosis statistics range of results was quite narrow (−0.489 to 0.926) (see Table 1). Moreover, we did not find statistically significant differences by country in the total scores of the general self-efficacy scale (t = 0.855; df = 129; *p* = 0.394), intention to get involved in the community (t = −1.577; df = 124; *p* = 0.117), participation in parents’ associations (t = −0.433; df = 128.975; *p* = 0.666), or in COmmuNity iNtervention SElf-Efficacy SCale for ParenT LEaDers (CONNECTED) (*t* = −1.054; *df* = 118; *p* = 0.294).

### 3.2. Internal Consistency and Reliability

The internal consistency was measured with Cronbach’s alpha coefficients; values from 0.70 to 0.80 were considered as being good, and those from 0.80 to 0.90 as very good [23]. Pearson’s correlation tests were used to calculate the intercorrelations between the scale and general self-efficacy, intention to get involved in the community, and parents’ association participation.

Regarding internal consistency using Cronbach’s alpha, the value of 0.899 observed indicated that the scale showed a very good reliability [24]. The two halves reliability was also good. In Table 2, Cronbach’s α results and the split-half estimates are shown.

Finally, Table 3 shows the test-retest stability and precision of the aggregate construct across the time results.

### 3.3. Validity

Convergent validity refers to how closely the scale of self-efficacy for community involvement is related to other measures that assess the same aggregate constructs. We found moderate significant correlations between the developed scales and the four assessments. Correlations with the intention to get involved in the community are above 0.5 (see Table 4), indicating how closely self-efficacy is related to the intention to be involved in the community.

### 3.4. CFA with SEM

A structural equation model was also run in order to assess a four-factor model of parents’ self-efficacy: individual self-efficacy, community self-efficacy, community intervention actions, and community intervention awareness. For the confirmatory factor analyses (CFA) using a structural equation model (SEM), given that item scores did not adjust to a normal distribution, we used the Weighted Least Squares Mean and Variance (WLSMV) method in order to test the suitability of the structure. This method has been shown to be more effective with categorical variables with few categories [25,26]. We used several model fit indexes: Chi-Square (χ2), Comparative Fit Index (CFI), and Root Mean Square Error of Approximation (RMSEA) [27]. According to the suggested cut-off values, a good model fit is associated with a small and significant χ2, values around 0.90 for CFI, and a RMSEA below 0.10 [28]. However, for the ML method, more demanding approaches emphasized values of at least 0.95 for the CFI and TLI and 0.06 or less for the RMSEA [29]. Some authors state that the chi-square index is no longer relied upon as a basis for acceptance or rejection due to its sensitivity to sample size [26,30,31] and, therefore, the use of multiple fit indexes provides a more holistic view of the goodness of fit.

**Figure 1:**
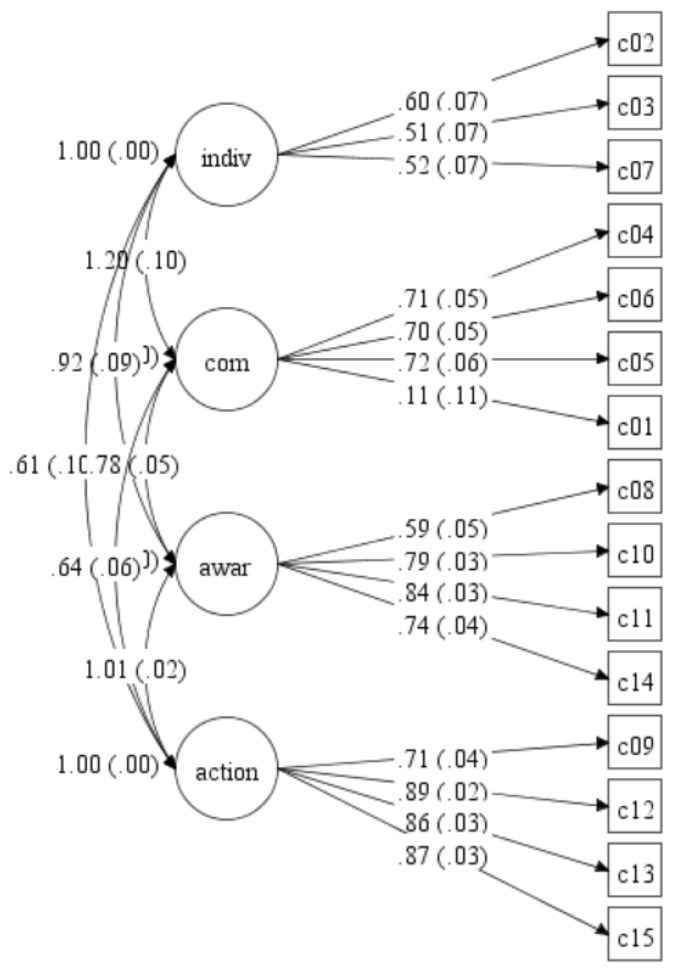
Confirmatory factor analysis with structural equation model. From left to right, the item number (inside the box), loadings of the items of the questionnaire (in the center of the arrows), and covariation among the latent constructs (the arrows connecting the spheres) are shown for the four-factor model. Comparative fit index = 0.90; root mean square error of approximation = 0.16; chi-square = 373.227; degrees of freedom = 84. Note: indiv = individual self-efficacy; com = community self-efficacy; awar = community intervention awareness; action = community intervention actions.

The results of the four-factor model showed an acceptable fit to the data. Although the chi-square index was statistically significant, other goodness of fit indices showed good values or values almost in the cut-off: the CFI obtained a value of 0.90, the TLI a value of 0.88, and the SRMR a value of 0.09. However, the RMSEA value of 0.16 indicated a poor fit. The four factors (individual self-efficacy, community self-efficacy, community awareness activities, and community actions) as well as the loadings of the items in each factor are presented in Figure 1.

## 4. Discussion

The aim of this article is to describe the development and evaluation of a new assessment tool to measure the degree of empowerment in parents who participate in in-school parent associations aimed at engaging their communities in actions to address risky alcohol use in adolescents. Since empowerment is both confidence in one’s own resources and tangible opportunities to generate influence within the community [3], we developed a scale to measure community self-efficacy, namely the COmmuNity iNtervention SElf-Efficacy SCale for ParenT LEaDers (CONNECTED). The internal consistency value of the scale suggests a good reliability. Regarding validity, the correlation between the scale and other tools assessing similar constructs was good. The CFA showed acceptable results, considering the limited sample size. All in all, the preliminary findings from our study seem positive in demonstrating that parents’ self-efficacy to engage in community actions can be reliably assessed using quantitative techniques. This is an important achievement because it will help assess the effectivity of empowerment interventions aimed to increase parents’ empowerment when they are in community networks, a line of environmental prevention which is increasingly the focus of research in recent years.

The scale being presented in this paper was developed within the context of the evaluation of the EPOPS project (Empowering Parents’ Organizations to Prevent Substance Use). The EPOPS program presents a bottom-up prevention approach to mobilize parents and resources where local authorities, for several reasons, are not able to do so. This situation is consistent in most Spanish and Portuguese regions and is comparable to the situation that most Southern European countries face.

Our behavior and socialization are very much driven by the regulatory, physical, and economic environment; the availability of alcohol; the ease of opportunity for unhealthy behaviors; and the perceived normality and acceptance of alcohol use. Alcohol use is common in adolescents’ environments in many European countries, and families can serve as a very important protective environment. However, although there is evidence that parents’ individual behaviors may reduce adolescents’ alcohol and substance use (e.g., clear, simple and neutral rules about the non-use of substances, or the supervision of adolescents’ leisure time or sharing family dinners), there are also other ways parents can influence other levels of the environment. In this framework, the Families in Network and Active (FERYA, in Spanish) and subsequently the EPOPS program were built from an exhaustive study carried out in six European countries [32,33] which showed that parenting styles work similarly across countries. The program offers parents an innovative way to be actively engaged in prevention, thus influencing the economic, physical, and regulatory environments that have a potential impact on adolescents’ behaviors. In the current economic climate, where local administrations may be influenced by the leisure or alcohol industries, well-organized groups of parents may sometimes be the only stakeholders in civil society that could have an impact on decision makers, thereby protecting the development of adolescents’ health and social behavior.

Some authors suggest that alpha values between 0.9 and 0.95 are excellent and higher than 8 is good [34,35,36]. Huh and colleagues [37] indicate that alpha values should be between 0.7 and 0.8 in confirmatory studies. Taking into account these cut-offs, our scale showed a good reliability.

Families are an important social agent, especially when they associate and foster changes at the community and environmental levels in partnership with other social agents, such as policy and decision makers, mass media, or industry representatives. Whereas a lack of empowerment results in helplessness and dependency, high levels of parental empowerment result in resilience and confidence in decision-making and proactive behavior [5]. A few examples of empowerment assessment tools that have been applied to specific fields such as users of mental health services [36] or families with children with emotional disabilities [9] can be found. The strength of this study is that it addressed both the design and the evaluation of a parent empowerment scale to assess parents’ engagement in community actions.

However, a number of limitations have to be mentioned. First, due to the number of items of the questionnaire and the fact that some items are very similar, the alpha obtained in the analysis could have been increased artificially. Sample sizes for future studies need to be larger. Some authors [38] indicate that increasing the sample size or increasing the number of indicators would affect the power of the results. This may be the case with this questionnaire, in which we have obtained a tendency for goodness of fit in some indices despite bad results in measures affected by sample size. We think that increasing the sample in future studies would help to better assess the quality of the questionnaire. Besides this, increasing the sample would allow us to randomly split the sample and perform EFA on one split and CFA on the other, as some authors suggest [39]. Second, the sample was composed of parents engaged in in-school parents’ associations. Therefore, they might have characteristics different to those of parents not engaged in such organizations. As we did not measure the socio-economic factors and the participants were non-randomly recruited, potential social factors of empowerment may have influenced our findings. Future studies should aim for a more heterogeneous sample, including parents who are not members of parents’ organizations. This would then enable the comparison of empowerment in parents who are not part of parents’ associations to those who are. The studies should also control for socio-economic variables. Measurement invariance regarding gender and/or nationality should also be assessed. On the other hand, we assumed parents’ leadership because they belonged to and actively worked in the parents’ association. However, in some cases, parents may have felt compelled to take up these positions, especially if no one else was willing to do so. Future studies may assess the degree to which parents freely chose to have active positions in their associations and evaluate if this is related to their empowerment or self-efficacy. Moreover, the validity of the questionnaire may have been influenced by social desirability. Future studies should use some measures to control for this potential bias. Finally, parental engagement in community life carries strong cultural influences. Taking into account that the CONNECTED Scale has been designed and validated in Spain and Portugal, its use in other cultures, such as Northern European countries, will require a process of cultural adaptation that takes into account the way in which parents are involved in the community.

## 5. Conclusions

In conclusion, our findings present preliminary results indicating that parent self-efficacy to engage in community actions can be assessed with the new scale developed. We believe that it captures the core features of parent community self-efficacy and hope that parent empowerment could be examined in the future using this scale to replicate our findings in different populations of parents and settings for the work of parent associations.

Environmental and community prevention strategies supported by families to influence decision-making and improve their social environment constitutes a promising preventive approach, although assessment tools are needed in this field. The developed scale could be a first step to identify areas of need within a community, and to monitor the progress and evaluate the outcomes of the preventive interventions implemented.

## Figures and Tables

**Table 1:** Means, standard deviations, adjusted item-total correlation, skewness, and kurtosis for all items (range: 1–5).

Items	N	Mean (SD)	Adjusted Item-Total Correlation	Skewness (SE)	Kurtosis (SE)
C01. I have control over decisions that affect my life	120	3.94 (0.811)	0.451	−0.327 (0.212)	−0.489 (0.420)
C02. My community has influence over decisions that affect my life	120	3.14 (0.982)	0.496	−0.589 (0.211)	−0.088 (0.419)
C03. I am satisfied with the degree of control I have over the decisions that affect my life	120	3.80 (0.836)	0.549	−0.634 (0.211)	0.449 (0.419)
C04. I can influence the decisions that affect my community	120	3.33 (0.907)	0.582	−0.575 (0.212)	0.105 (0.420)
C05. Working together, people in my community can influence the decisions that affect us	120	4.01 (0.824)	0.611	−0.845 (0.211)	0.996 (0.419)
C06. People in my community collaborate to influence decisions at the local, regional or national level	120	3.11 (0.946)	0.433	−0.505 (0.211)	−0.409 (0.419)
C07. I am satisfied with the degree of influence I have on the decisions that affect my community	120	3.21 (0.903)	0.526	−0.565 (0.212)	−0.064 (0.420)
C08. To prevent street drinking in my neighborhood	120	3.01 (0.924)	0.620	−0.142 (0.217)	0.129 (0.431)
C09. To sensitize mothers and fathers of the need to put pressure on the authorities to eliminate or reduce the consumption of young people and adolescents	120	3.39 (0.870)	0.743	−0.334 (0.217)	0.205 (0.430)
C10. To make the changes my community needs	120	3.24 (0.797)	0.695	−0.072 (0.217)	0.754 (0.430)
C11. To improve my community or neighborhood	120	3.27 (0.766)	0.726	−0.055 (0.217)	0.612 (0.431)
C12. To negotiate with the authorities to get improvements in my community	120	3.32 (0.903)	0.695	−0.214 (0.217)	−0.137 (0.430)
C13. To influence the other members of my association of mothers and fathers to be involved in community actions	120	3.43 (0.817)	0.694	−0.319 (0.217)	0.311 (0.430)
C14. To promote changes that improve my community or neighborhood.	120	3.38 (0.820)	0.756	−0.530 (0.217)	0.926 (0.430)
C15. To influence people around me (friends, family, work …) to be involved in community actions	120	3.46 (0.809)	0.668	−0.486 (0.217)	0.878 (0.430)

**Table 2:** Cronbach’s alpha and split-half estimates of the scale.

Scale	N	Items	Mean (SD)	Range	Cronbach’s α	Two Halves (Spearman-Brown)
COmmuNity iNtervention SElf-Efficacy SCale for ParenT LEaDers (CONNECTED)	120	15	50.94 (8.30)	24–70	0.899	0.734

**Table 3:** Temporary stability (intraclass correlation coefficient).

Scales	N	Spearman’s Correlation
General Self-Efficacy Scale	29	0.804 **
Intention to get involved in community	28	0.675 *
Parent’s Association Participation	30	0.821 **
COmmuNity iNtervention SElf-Efficacy SCale for ParenT LEaDers (CONNECTED)	28	0.669 *

“*” *p* < 0.05, “**” explanation < 0.001.

**Table 4:** Convergent validity: Spearman correlation and *n*.

Scales	CONNECTED	GSES	Intention	PAP
COmmuNity iNtervention SElf-Efficacy SCale for ParenT LEaDers (CONNECTED)	1	0.572 ** (*n* = 119)	0.531 ** (*n* = 114)	0.408 ** (*n* = 120)
General Self-Efficacy Scale (GSES)		1	0.248 ** (*n* = 126)	0.220 * (*n* = 131)
Intention to get involved in community (Intention)			1	0.408 ** (*n* = 126)
Parent’s Association Participation Questionnaire (PAP)				1

“*” *p* < 0.05, “**” explanation < 0.001.
